# TNF induced inhibition of *Cirbp* expression depends on RelB NF-κB signalling pathway

**DOI:** 10.1016/j.bbrep.2015.11.007

**Published:** 2015-11-14

**Authors:** Martin A. Lopez, Daniel Meier, W.Wei-Lynn Wong, Adriano Fontana

**Affiliations:** Institute of Experimental Immunology, University of Zurich, 8057 Zurich, Switzerland

**Keywords:** Circadian rhythm, Cytokine, Gene transcription, Inflammation, Signalling pathway

## Abstract

The circadian clock is required for the rhythmic expression of a plethora of genes that orchestrate metabolism, sleep-wake behaviour and the immune response to pathogens. The cold-inducible RNA binding protein (CIRBP) is required for high amplitude expression of clock genes. Moreover, CIRBP protects the expression of clock genes from the inhibitory effects of tumour necrosis factor (TNF). However, since TNF represses *Cirbp* expression, the protective effect of CIRBP is lost. Here, we show that the TNF effect on *Cirbp* requires the non-canonical NF-κB signalling pathway. While a knock down of RelA does not alter the effects of TNF on *Cirbp,* a knock down of RelB represses this effect. In addition, the data indicate that p50 and p52 are required in the TNF induced inhibition of *Cirbp*. These results show that *Cirbp* expression in TNF treated cells is regulated via the non-canonical NF-κB pathway.

## Highlights

• TNF effect on *Cirbp* expression is inhibited by blocking IKKα or IKKβ.
• Knockdown of RelB prevents TNF dependent *Cirbp* repression.
• Non-canonical NF-κB pathway seems to regulate *Cirbp* expression in inflammation.

## 1 Introduction

Circadian rhythms are mediated by clock genes, and thereby regulate metabolism and sleep-wake behaviour [1], [2]. The main transcription factors coordinating the circadian rhythms are CLOCK and BMAL1, which form heterodimers and activate the gene expression of *Period* (*Per*), *Cryptochrome* (*Cry*) and of various clock controlled genes by binding to E-box motives [3]. Besides, CLOCK can be substitute by its paralog NPAS2 [4]. The binding of CLOCK:BMAL1 to the E-box is regulated by positive and negative feedback mechanisms [3].

Recent studies point to a crosstalk between the clock and the immune system. In mice it is well established that the response to various pathogens and to pro-inflammatory cytokines is under circadian control. Hence, the immune system of mice is especially sensitive to pathogens at the beginning of their active phase [5]. The macrophage response to lipopolysaccharide (LPS) is decreased in mice with an inactive *clock* gene [6]. The extent of secretion of tumour necrosis factor (TNF) and interleukin (IL) -6 by LPS stimulated macrophages follows a circadian rhythm [7]. A defect in *Per2* expression affects Toll-like receptor (TLR) 9 expression and thus the vaccine response, when using TLR9 ligands as adjuvants [8]. Besides of effects of the circadian system on the immune response, the immune system also influences the circadian clock. TNF inhibits the expression of all three *Period* genes and of the PAR-bZip transcription factors, *Dbp, Tef* and *Hlf* [9]. At least part of these effects, are due to an interference with the E-box dependent transcription [9]. Additionally, transforming growth factor beta (TGFβ) has been shown to reduce the expression of several clock genes, including *Per1, Per3, Dbp* and *Tef* [10], [11]. The effect of TGFβ is mediated by induction of *Dec1* [11].

A recent study demonstrated that the cold-inducible RNA binding protein (CIRBP, also called CIRP and hnRNP A18) interacts with transcripts associated in circadian behaviour and that CIRBP is required for high amplitude expression of clock genes [12]. CIRBP was originally identified as the first mammalian cold shock protein [13] and has been shown to be upregulated by various cellular stresses, including hypoxia and UV-irradiation [14], [15], [16]. CIRBP belongs to the glycine rich RNA binding protein family and is thought to modulate gene expression by stabilising transcripts through binding to the 5′- untranslated region (UTR) or 3′- UTR [12], [17], [18]. CIRBP is released by necrotic cells and thereby is found in the serum of patients with hemorrhagic shock and sepsis [19]. By binding to TLR4, CIRBP activates the inflammasome and promotes the production of proinflammatory cytokines, including TNF and IL-6 [19]. Moreover, CIRBP has been reported to link inflammation and tumorigenesis in colitis-associated cancer and to increase reactive oxygen species (ROS) accumulation and CD133, thereby leading to enhanced liver tumorigenesis [20], [21].

Recently, we have demonstrated that TNF not only interferes with E-box mediated expression of clock genes, but also impairs the expression of *Cirbp* in fibroblasts and neuronal cells [22]. The inhibitory effect of TNF on clock genes is more pronounced in *Cirbp* knock out cells. On the other hand overexpression of CIRBP protects clock genes from the TNF effect [22]. These data indicate that CIRBP counteracts the inhibitory effect of TNF on clock gene expression. In the light of the role of TNF to exert many of its effects by activating the NF-κB pathway, we have analysed the involvement of this signalling pathway in the TNF mediated inhibition of *Cirbp* expression.

In mammals, the NF-κB family comprises five different subunits, belonging to the Rel family, which can either hetero- or homodimerise in order to form transcriptionally active isoforms. These isoforms can have very different roles in the transcriptional activation or repression of inflammatory genes [23]. The signalling pathways that mediate NF-κB activation can be classified into canonical and non-canonical (or alternative) pathways. In the canonical pathway, RelA (also known as p65) and p50 form heterodimers in order to activate gene expression. In a non-active form this complex is bound to the inhibitory protein IκB which keeps the heterodimer in the cytoplasm. Once IκB undergoes phosphorylation and subsequently degradation the activated p65::p50 complex translocates into the nucleus, binds to its consensus sequences and activates the gene expression of its target genes [24]. The activation of the non-canonical NF-κB pathway involves different signalling molecules and leads to the predominant activation of the p52::RelB dimer [25]. RelB itself is very labile and requires the initial binding of p100 [26], the precursor protein of p52. Processing of p100 generates p52 and leads to the translocation of the p52::RelB dimer into the nucleus [25], [27].Although p100 preferentially binds RelB, RelB can also form heterodimers with p50 after IκB degradation. This complex is also able to translocate into the nucleus but this pathway is less understood [28].

Here, we show that inhibition of the NF-κB signalling prevents from the TNF mediated suppression of *Cirbp* expression. Our data point to the involvement of the non-canonical pathway in the TNF effect on *Cirbp.*

## 2 Material and methods

### 2.1 Treatment of cells with TNF and NF-κB inhibitors

NIH3T3 fibroblasts (DSMZ), were grown in DMEM high glucose (4,5 g/l) medium (Gibco) supplemented with 10% FCS (PAN Biotech) and gentamicin (1x; Gibco). Cells were kept at 37 °C and 5% CO_2_. For RT-qPCR analysis, cells were seed in triplicates in 12-well plates in a density of 1×10^5^ per well. For Western Blots cells were seed in T25 flasks in a density of 1×10^6^ per flask. Two days after seeding, cells were synchronized by serum deprivation (1% FCS). Whereas the NF-κB inhibitors IKK III (Calbiochem 401480) and IKK VII (Calbiochem 401486) were added immediately after serum deprivation, TNF (10ng/ml) (Peprotech) was added one hour later. The concentrations we used for the blockers of the NF-κB signalling pathway have already been described to be effective [29], [30]. Isolation of RNA and protein was performed in cells cultured for 4 and 6 h respectively. The effect of the inhibitors was assessed in cultures treated for 3 h in the presence or absence of TNF.

### 2.2 Treatment of cells with siRNA against p50, p52, RelB and RelA

NIH3T3 cells and primary fibroblasts were seeded in 6-well plates (1.5×10^5^). After 24 h they were transfected with DharmaFECT 1 transfection reagent (Dharmacon) and 25 nM of siRNA (Dharmacon) against *p50, p52* or *RelB. Gapdh* and no target siRNA were used as positive and negative controls, respectively. The siRNA targeting *RelA* was obtained from QIAGEN. After transfection cells were incubated for 36 h for RNA analysis and 48 h for protein analysis.

### 2.3 RNA isolation and gene expression analysis

Whole-cell RNA from cultured cells was extracted using NucleoSpin-RNA II kit RNA (Machery Nagel) according to protocol. Subsequently, 1 µg RNA was reverse-transcribed using random hexamers (Fermentas) and M-MuLV reverse transcriptase (Life Technologies). 20 ng of cDNA was amplified in a CFX384 detection system (Biorad) using the TaqMan precision PLUS Master mix (Primerdesign). The gene expression level was normalised to three housekeeping genes (*eEF1a1, HPRT*, *GAPDH*) using qBase software [31]. Each CT value used for these calculations is the mean of duplicates of the same reaction. Relative RNA levels are expressed as percentages of the average control groups. Taqman primers were obtained from Primerdesign and Life Technologies.

### 2.4 Analysis of CIRBP by Western Blot

Cells were lysed with the IP lysis buffer (Pierce) as described in the protocol. Whole protein extracts (50 µg) in LDS sample buffer (Invitrogen) and DTT were applied on a NuPAGE 12% Bis-Tris-Gel (Invitrogen). The proteins were separated at constant 150 V in a MES SDS running buffer (Invitrogen). Subsequently, blotting on a PVDF membrane was performed in a full wet tank blot at 30 V. Membranes were incubated with a CIRBP rabbit polyclonal antibody recognising the C terminus of mouse CIRBP [32]. As secondary antibody an HRP-conjugated goat anti-rabbit (ab79051, Abcam) was used. As a loading control, an antibody to the mouse nuclear matrix protein p84 was used (ab487); with a goat to mouse HRP (ab97023) secondary antibody. The p105/p50 antibody (ab7917) was from Abcam and the IκB antibody (9242) from Cell Signaling Technology. Densitometric analysis was done using ImageJ software. The protein marker used was the prestained protein ladder V (PL005) from Geneaid.

### 2.5 Statistics

All experiments were performed in biological triplets and analysed with GraphPad Prism (GraphPad Software). To assess the statistical significance between single series of measurements we used the two-tailed Student’s *t*-test. For grouped measurements with one variable for the selected genes, a one-way ANOVA was used, followed by a Bonferroni post-hoc test to decrease the likelihood of a rare event in multiple comparisons. *P* values less than 0.05 were considered significant, 0.01 in ANOVAs with post-hoc tests. Data are shown as mean +/− SEM of triplicate cultures.

## 3 Results

### 3.1 Inhibition of NF-κB signalling pathway interferes with TNF mediated suppression of Cirbp expression

**Fig. 1:**
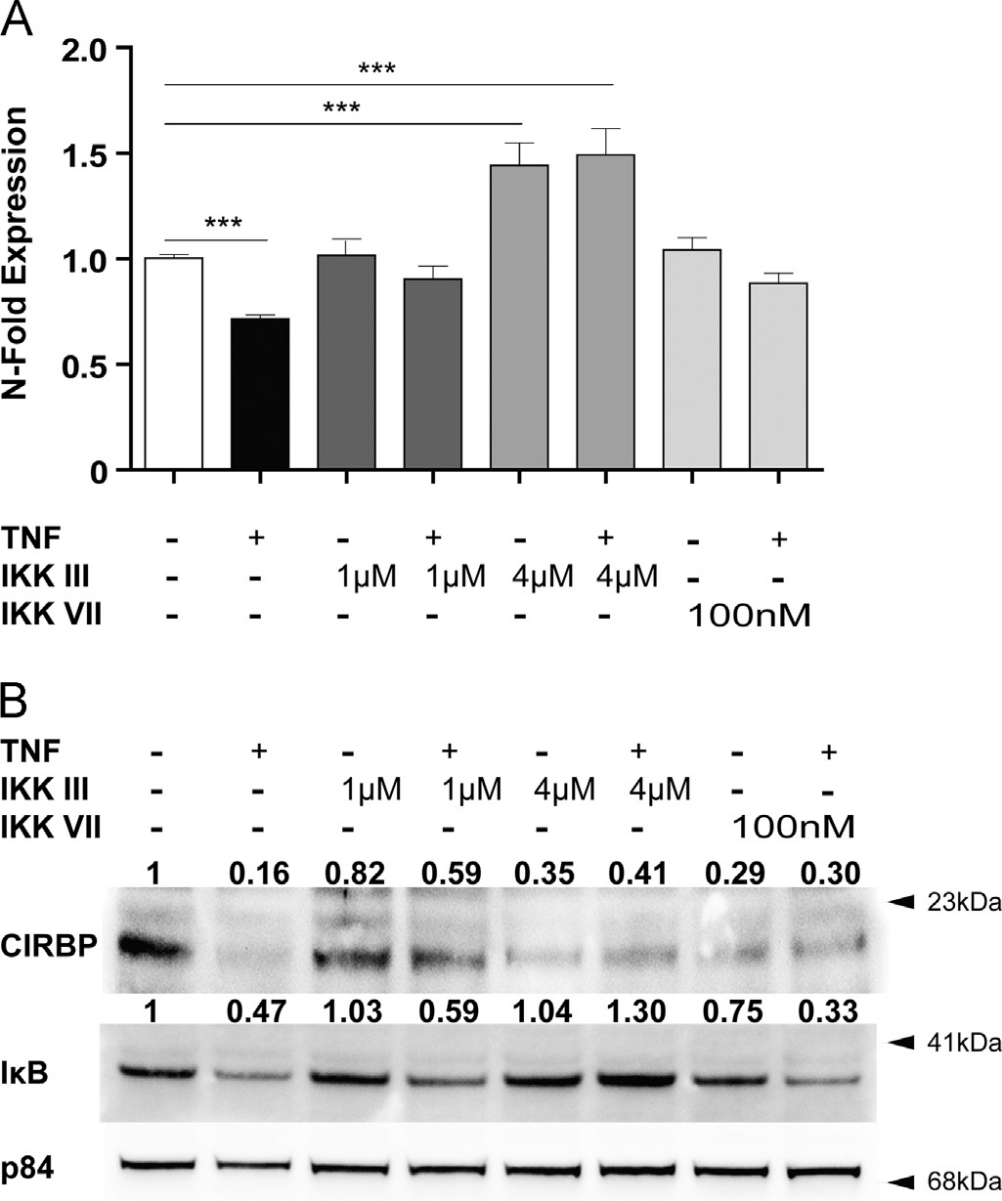
Inhibition of IKKα and IKKβ prevents TNF mediated inhibition of Cirbp expression. (A) NIH3T3 cells were treated with the IKKα and β inhibitors IKK III and IKK VII in the presence or absence of TNF (10 ng/ml). Data show the expression of Cirbp mRNA (A) and protein (B). As a control for the effectiveness of the NF-κB blockers the expression of IκB was used, Data of RT-qPCR assays show the mean +/− SEM (error bars) of biological triplicates from three independent experiments. Significance of grouped results were calculated with one way-ANOVA and Bonferroni post-hoc test; ^⁎⁎⁎^*p*<0.001. Western blots were quantitated by densitometric analysis; the respective data are given above the western blots.

We have recently reported that treatment of NIH3T3 cells with TNF reduces the expression of *Cirbp* [22]. To assess the involvement of the NF-κB pathway we blocked this pathway by using IKK III and IKK VII, the compounds being well described to interfere with the effects of IKKα and IKKβ, respectively [30], [33]. As shown in Fig. 1A TNF inhibits the expression of *Cirbp* by 29%. Treatment with IKK III (1 µM) reduced the extent of inhibition to 10%. The increase of IKK III concentrations to 4 µM enhanced basal expression of *Cirbp* and blocked the inhibitory effect of TNF. IKK VII in concentrations of 100 nM was sufficient to prevent any significant inhibitory effect of TNF on *Cirbp*. As described recently the effect of TNF on *Cirbp* expression is also seen at the protein level (Fig. 1B) [22]. When adding IKK III (1 µM) the extent of TNF induced inhibition was reduced. Unlike the basal expression of *Cirbp* mRNA the expression of CIRBP protein was not enhanced when adding IKK III at higher concentrations (4 µM). Still, also at this concentration IKK III interfered with TNF mediated inhibition of CIRBP. The effects of IKK VII resembled those seen with the higher concentrations of IKK III, namely inhibition of basal expression of CIRBP and prevention of the inhibitory effect of TNF (Fig. 1B). To assess the effectiveness of IKK III and IKK VII we used IκB as a positive control. Whereas higher concentrations of IKK III effectively prevented from the suppression of the TNF mediated inhibition of IκB, IKK VII was less effective.

### 3.2 Regulation of Cirbp expression via the non-canonical NF-κB signalling pathway

**Fig. 2:**
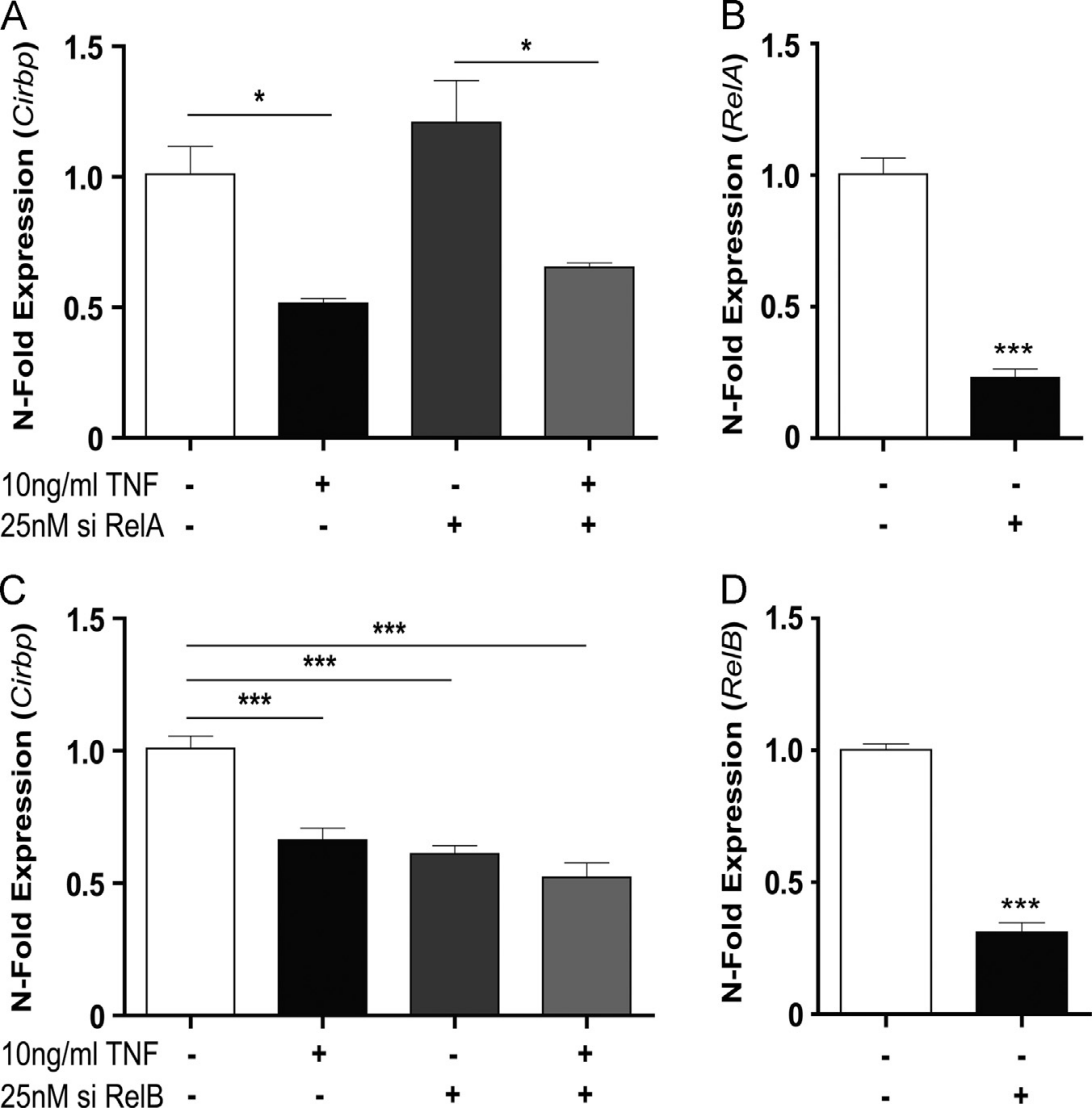
Knockdown of RelB, but not of RelA prevents from the inhibitory effect of TNF on Cirbp expression. Cells transfected with siRNA against RelA (A and C) or siRNA against RelB (B and D) were left untreated or treated with TNF (10 ng/ml) for 4 h. Data show the expression of Cirbp (A and B) and the effectiveness of siRNA treatment against RelA (C) and RelB (D). Knockdown reduces RelA expression by 77% and RelB by 70%. Data of RT-qPCR assays show the mean +/−SEM (error bars) of biological triplicates from three independent experiments. Significance of grouped results were calculated with one way-ANOVA and Bonferroni post-hoc test; significance for siRNA effectivity was calculated with a two-tailed Student's *t*-test; ^⁎^*p*<0.01 ^⁎⁎⁎^*p*<0.001.

In the light of the well described effect of TNF to activate the canonical NF-κB signalling pathway we knocked down *RelA* expression in NIH3T3 cells using siRNA. Surprisingly, the downregulation of *RelA* did not protect the cells from TNF mediated inhibition of *Cirbp* expression (Fig. 2A). Hence, we used siRNA targeting *RelB* to analyse whether the TNF effect on *Cirbp* might require the non-canonical NF-κB signalling pathway. A knock down of *RelB* in NIH3T3 cells reduced basal expression of *Cirbp* and prevented from the inhibitory effect of TNF on *Cirbp* (Fig. 2B). Effectiveness of siRNA treatment was assessed by controlling the expression of *RelA* and *RelB* in transfected cells (Figs. 2C and D). Taken collectively, the data indicate that the TNF effect on *Cirbp* involves the non-canonical NF-κB signalling pathway.

### 3.3 Knock down of p50 or p52 seem to inhibit the TNF effect on Cirbp

**Fig. 3:**
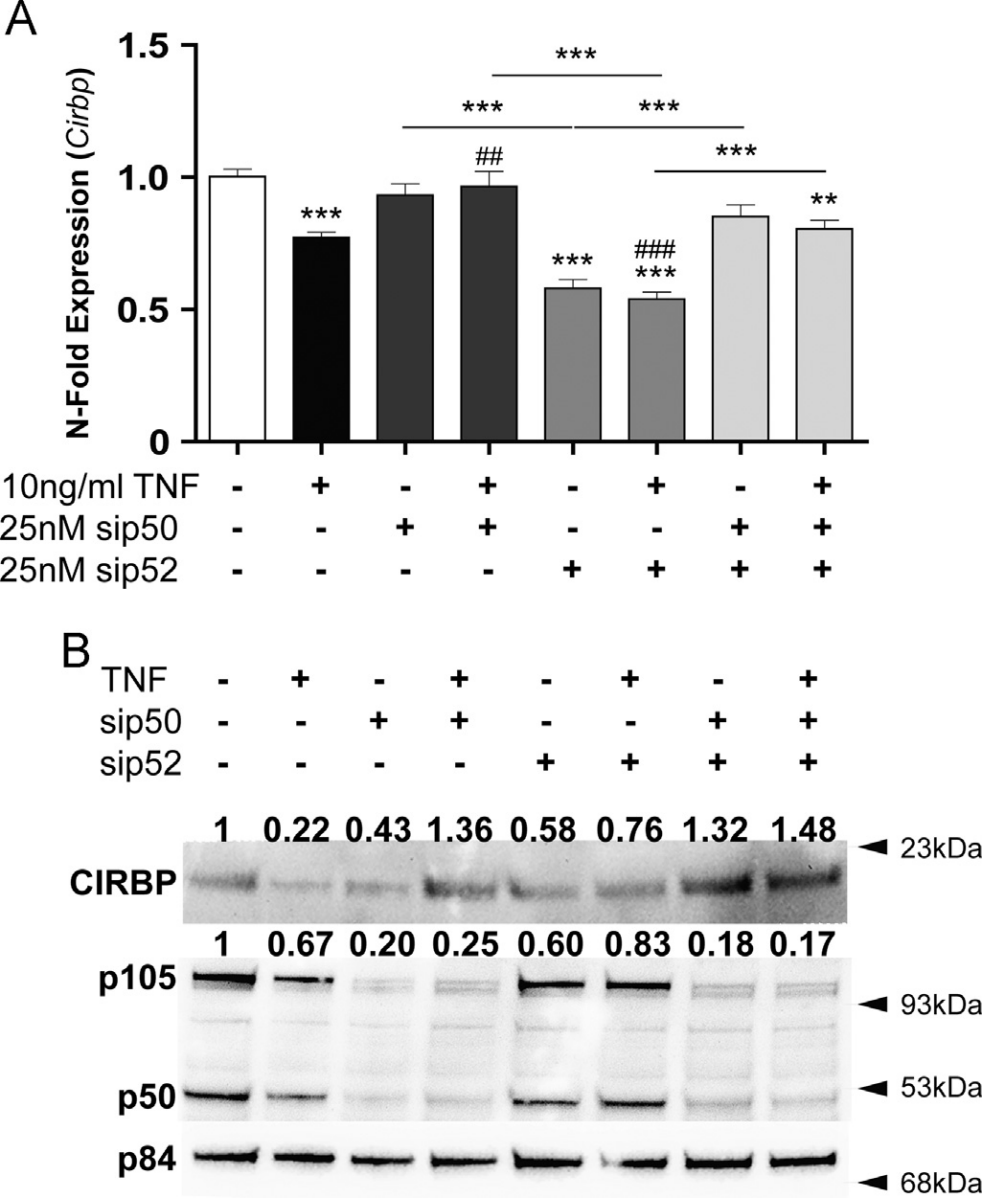
Knockdown of *p50* and *p52* reduces the TNF induced inhibition of Cirbp expression. NIH3T3 cells were transfected with siRNA against *p50*, *p52* or both. Thereafter, cells were treated with TNF (10 ng/ml). Expression of mRNA (Fig. 3A) and protein (Fig. 3B) was assessed after 4 h and 6 h of TNF treatment, respectively. Compared to nontransfected control cells knockdown of *p52* reduces *Cirbp* mRNA expression by 42%. Densitometric analysis shows data for CIRBP and *p50*. Since the antibodies to *p52* proved not to work reliably, the respective data are not included. Data of RT-qPCR assays show the mean +/− SEM (error bars) of biological triplicates from four independent experiments. Significance of grouped results were calculated with one way-ANOVA and Bonferroni post-hoc test; ^⁎⁎^*p*<0.01, ^⁎⁎⁎^*p*<0.001; ^⁎^ without line significance compared to untreated NIH3T3; # gives significance compared to NIH3T3+TNF.

Since the knock down of *RelB* reduces the inhibitory effect of TNF on *Cirbp* expression*,* we assessed the involvement of RelB’s binding partners in the TNF effect observed. RelB preferentially binds to p100/p52, but also forms heterodimers with p50 [28]. As shown in Fig. 3A, TNF failed to reduce the expression of *Cirbp* in NIH3T3 cells transfected with siRNA against *p50* or against *p52*. Analogously, the knockdown of both genes mimicked the aforementioned effects. Effectiveness of siRNA treatment was again assessed by controlling the expression of *p50* and *p52* in transfected cells (Fig S1). Western blots show the knockdown of p50 and of p52 to reduce the basal expression of CIRBP, an effect not being seen in NIH3T3 cells transfected with siRNA against both genes (Fig. 3B). The inhibitory effect of TNF was not observed in cells transfected with siRNA against *p50* or against both *p50* and *p52*. A single knockdown of *p52* was less effective.

## 4 Discussion

The data presented here shows that the NF-κB signalling pathway plays a pivotal role in the previously described effect of TNF to inhibit the expression of *Cirbp* [22]. In a first approach, we used the NF-κB inhibitors IKK III and IKK VII, which both have been well described to interfere with NF-κB activation. IKK III has been described to block cytokine production, to interfere with TNF mediated expression of adhesions receptors in endothelial cells and supress the immune response. IKK VII has been described as a potent IKKβ inhibitor being even effective in low-nanomolar range [29], [30], [34]. Both inhibitors effectively blocked the inhibitory effect of TNF on *Cirbp* expression. When exploring the involvement of individual components required for NF-κB activation by using siRNAs, we identified RelB, rather than RelA to be essential for TNF mediated inhibition of *Cirbp*. Thus, TNF effects on *Cirbp* are mediated via the non-canonical pathway. This is especially interesting since TNF is known to activate the canonical NF-κB pathway rather than the non-canonical one. This has been worked out in different biological systems. For example, mice lacking RelA loose the protection from TNF induced apoptosis [35]. The TNF mediated induction of the leukaemia inhibitory factor (LIF) is abolished in *relA* knock out cells [36]. In contrast, RelB has been described to repress *Tnf* mRNA expression by epigenetic silencing the promotor [37]. Further, TNF has been shown to induce p100, which in return inhibits RelB::p50 complexes [38]. Since RelB forms heterodimers we assessed the role of its main binding partner p52. Indeed, siRNA against *p52* were effective in prevention of inhibitory TNF effects on *Cirbp*. TNF stimulation has been described to induce a delayed processing of p100 to form p52 thereby leading to a postponed activation of the non-canonical NF-κB pathway [39]. On the other hand, we find that besides of the contribution of RelB and p52, also p50 to be essential in the TNF effects studied. A knock down of *p50*, which forms heterodimers with RelA, leads also to an inhibition of the TNF effect on *Cirbp.* However, p50 can also form heterodimers with RelB. This complex is also able to induce gene expression [28]. Besides, p50 homodimers are known to block the expression of *Tnf* mRNA [40].

CIRBP has been shown to be required for high amplitude expression of clock genes [12]. Further, the TNF dependent downregulation of clock genes has been shown to be dependent on *Cirbp* expression [22]. However, the downregulation of *Cirbp* only plays a role in inhibition of clock gene expression when adding TNF at low doses (1 ng/ml). When using higher TNF concentrations downregulation of clock genes was found to depend on TNF induced induction of Twist1 [41]. Twist1 was found to interfere with CLOCK mediated activation of E-boxes in the promoter regions of clock genes. In the light of the data on TNF effects on *Cirbp* expression to depend on NF-κB activation, it is interesting that also the upregulation of Twist1 by TNF is mediated through NF-κB signalling [42], [43]. Hence, the NF-κB signalling pathway might be the main regulator of clock gene expression in inflammation.

In conclusion, we showed that TNF mediated downregulation of *Cirbp* requires several components of the NF-κB signalling pathway, namely RelB, p50 and p52, while RelA seems not to be required for this effect. Thus, this process appears to be regulated through the non-canonical NF-κB pathway.
