# Supplementary figures and images for: TNF induced inhibition of Cirbp expression depends on RelB NF-κB signalling pathway
Source: Biochem Biophys Rep. 2015 Nov 14;5:22–6. doi: 10.1016/j.bbrep.2015.11.007 (PMC5600431; doi:10.1016/j.bbrep.2015.11.007)

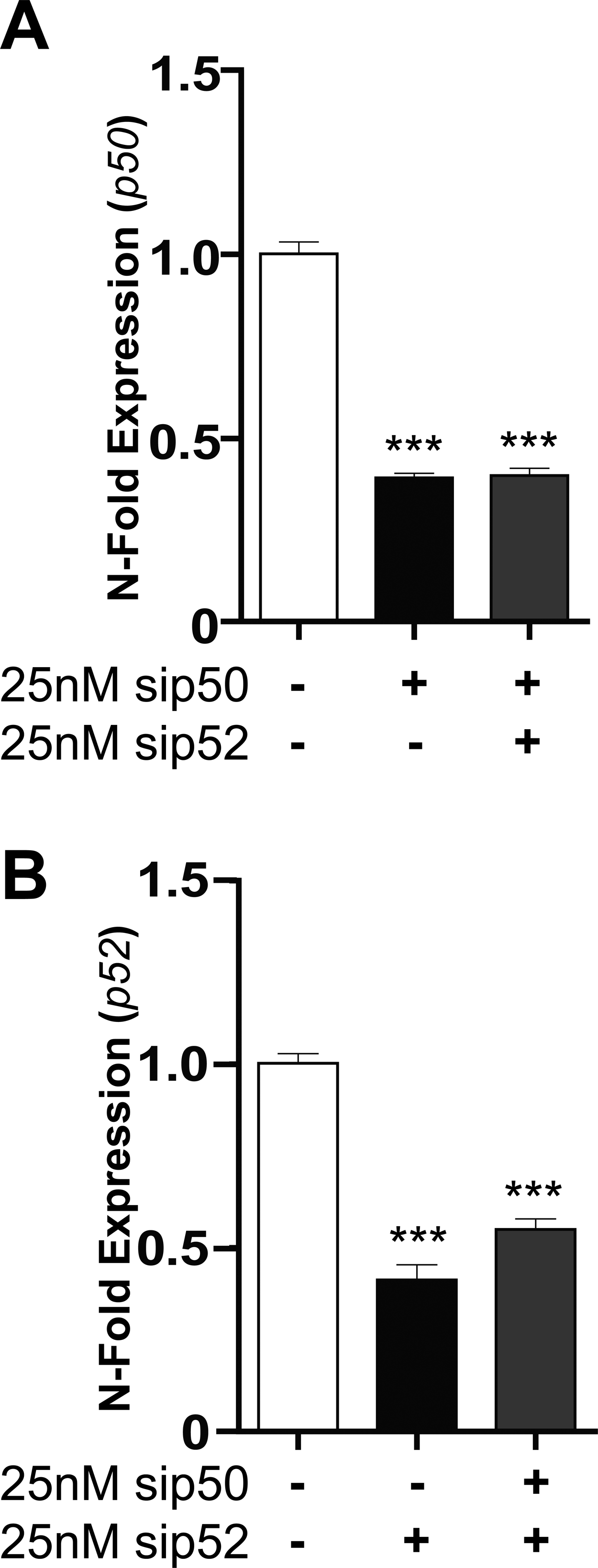

Supplement: Supplementary file 1 — Supplementary material [file mmc1.zip › mmc1.tif]
